# Determinants of *Campylobacter* species diversity in infants and association with family members, livestock, and household environments in rural Eastern Ethiopia

**DOI:** 10.1186/s13099-025-00725-0

**Published:** 2025-07-05

**Authors:** Amanda Ojeda, Loïc Deblais, Bahar Mummed, Mussie Brhane, Kedir A. Hassen, Belisa Usmael Ahmedo, Yenenesh Demisie Weldesenbet, Dehao Chen, Xiaolong Li, Cyrus Saleem, Mark J. Manary, Luiz F. W. Roesch, Sarah L. McKune, Arie H. Havelaar, Gireesh Rajashekara, the CAGED Research Team, Amanda Ojeda, Loïc Deblais, Kedir A. Hassen, Belisa Usmael Ahmedo, Yenenesh Demisie Weldesenbet, Dehao Chen, Xiaolong Li, Cyrus Saleem, Mark J. Manary, Sarah L. McKune, Arie H. Havelaar, Gireesh Rajashekara, Abadir Jemal Seran, Abdulmuen Mohammed Ibrahim, Bahar Mummed Hassen, Efrah Ali Yusuf, Getnet Yimer, Ibsa A. Ahmed, Ibsa Aliyi Usmane, Jafer Kedir Amin, Jemal Y. Hassen, Kunuza Adem Umer, Karah Mechlowitz, Kedir Teji Roba, Mussie Bhrane, Mawardi M. Dawid, Mahammad Mahammad Usmail, Nigel P. French, Nur Shaikh, Nitya Singh, Wondwossen A. Gebreyes, Yang Yang, Zelalem Hailu Mekuria

**Affiliations:** 1 University of Florida, Gainesville, FL USA; 2 Hypercell Technologies, Ithaca, NY USA; 3 Haramaya University, Dire Dawa, Ethiopia; 4 Washington University, St Louis, MO USA; 5 University of Illinois Urbana-Champaign, Champaign, IL USA

**Keywords:** *Campylobacter*, *C. infans*, *C. jejuni*, *C. upsaliensis*, qPCR, Longitudinal study, Eastern Ethiopia, Infant stool, Household determinants, Environmental enteric dysfunction (EED)

## Abstract

**Background:**

*Campylobacter* infections pose a significant challenge in low- and middle-income countries, contributing to child mortality. *Campylobacter* is linked to acute gastrointestinal illness and severe long-term consequences, including environmental enteric dysfunction (EED) and stunting. In 2018, our cross-sectional study in Ethiopia detected *Campylobacter* in 88% of stools from children aged 12–15 months, with an average of 11 species per stool using meta-total RNA sequencing. Building on these findings, we conducted a longitudinal study (December 2020–June 2022) to investigate *Campylobacter* colonization of infants and identify reservoirs and risk factors in rural eastern Ethiopia.

**Results:**

After a preliminary screening of 15 *Campylobacter* species using species-specific quantitative PCR, we analyzed four target species in 2045 samples from infants (first month to just one year of life) and biannual samples from mothers, siblings, and livestock (goats, cattle, sheep, and chickens). *Candidatus* C. infans (41%)*, C. jejuni* (26%), and *C. upsaliensis* (13%) were identified as the predominant in the infant gut. Colonization of *C. infans* and *C.jejuni* increased (*C. infans*: 0.85%, *C. jejuni*-0.98% increase/ day in the odds of colonization) and abundance (P = 0.027, 0.024) with age. Enteric symptoms were strongly associated with *C. infans* (diarrhea: OR = 2.02 [95%CI: 35%,100%]; fever: OR = 1.62 [95%CI: 14%, 83%]) and *C. jejuni* (diarrhea: OR = 2.29 [95%CI: 46%,100%], fever: OR = 2.53 [95%CI: 56%,100%]). Based on linear mixed models, we found elevated cumulative loads of *C. infans* load in infants (especially females OR = 1.5 [95%CI: 10%, 67%]), consuming raw milk (OR = 2.3 [95%CI: 24%,100%]) or those exposed to areas contaminated with animal droppings (OR = 1.6 [95%CI: 7%,93%]), while *C. jejuni* cumulative loads were higher in infants ingesting soil or animal feces (OR = 2.2 [95%CI: 23%,100%]). *C. infans* was also prevalent in siblings (56%) and mothers (45%), whereas *C. jejuni* was common in chickens (38%) and small ruminants (goats 27%, sheep 21%).

**Conclusions:**

*Campylobacter* was highly prevalent in rural Ethiopian infants. *C. infans* was primarily associated with human hosts, and *C. jejuni* was mainly linked to zoonotic sources. Our findings emphasize the need for targeted interventions addressing environmental, dietary, and behavioral factors to reduce *Campylobacter* transmission in resource-limited settings.

**Graphical Abstract:**

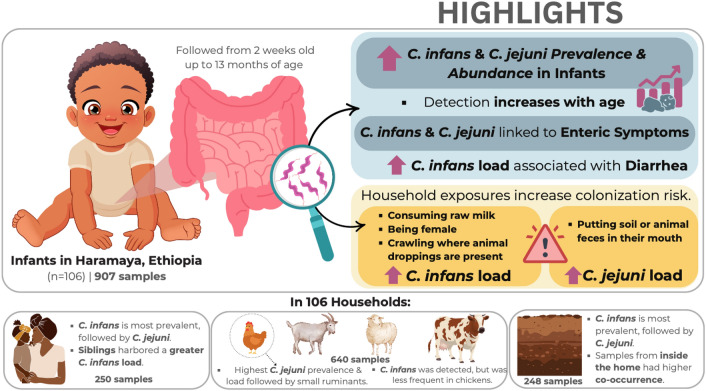

**Supplementary Information:**

The online version contains supplementary material available at 10.1186/s13099-025-00725-0.

## Background

*Campylobacter* infections represent a substantial global health concern. They have been identified as a leading cause of bacterial gastroenteritis, resulting in 166 million cases of diarrhea globally in 2020, of which 96 million were foodborne [1]. While most cases are self-limiting, the potential for severe, even life-threatening outcomes cannot be overlooked, particularly in infants, the elderly, and those with weakened immune systems [2].

*Campylobacter* infections in high- and middle-income countries are mainly foodborne [3], but other pathways are more important in settings with poor sanitation and hygiene infrastructure [4], contributing significantly to the disease spread. Beyond acute gastroenteritis, these infections have been linked to long-term gastrointestinal health issues, particularly in pediatric populations where the early-life gut microbiome is being established. This critical period is marked by rapid changes in the intestinal microbial community driven by diet and environment and generally stabilizes by three years of age [5]. Research from the Etiology, Risk Factors, and Interactions of Enteric Infections and Malnutrition and the Consequences for Child Health and Development (MAL-ED) project has associated *Campylobacter* infections with adverse health outcomes such as Environmental Enteric Dysfunction (EED) leading to compromised nutrient absorption, weakened immunity, and stunting [6–8]. These early-life growth patterns are crucial indicators of nutritional status, with significant implications for future health outcomes, including mortality, chronic disease, neurodevelopment, and economic productivity in later life [7–10].

Outbreaks of *Campylobacter* are rarely reported globally [11, 12]. Nevertheless, Africa is estimated to have the highest incidence of campylobacteriosis, particularly among children residing in both rural and urban areas in East Africa [13, 14]. In 2018, a formative cross-sectional study in rural eastern Ethiopia conducted under the CAGED project’s umbrella (*Campylobacter* Genomics and Environmental Enteric Dysfunction) unveiled that the prevalence of *Campylobacter* at the genus level was 88% in stool samples from children under two years of age [15]. Meta-total RNA sequencing revealed an average of 11 distinct *Campylobacter* species in positive stool samples from children, heightening concerns about their potential role in chronic outcomes such as EED and stunting [16].

Although by November 2024, 49 species have been validly published within the *Campylobacter* genus (https://lpsn.dsmz.de/genus/campylobacter), existing research disproportionately concentrates on thermophilic *Campylobacter jejuni* and *Campylobacter coli* [17]. *C. jejuni* is the dominant diarrhea-associated species worldwide, with *C. coli* contributing 1–25% to *Campylobacter*-related gastroenteritis cases [18, 19]. These species, while dominant, are not exclusive agents of *Campylobacter*-related gastroenteritis [20, 21]. Moreover, the MAL-ED study revealed that when detecting all *Campylobacter* species using immunoassays, the impact on stunting was greater than when looking at just *C. jejuni* and *C. coli* alone [7–9].

Most data on *Campylobacter* epidemiology are from high-income countries and it remains understudied in low- and middle-income countries, particularly the non-thermophilic species abundant in vulnerable populations like Ethiopian children enrolled in the CAGED formative study [15]. However, advancements in isolation and detection techniques have shed light on other emerging *Campylobacter* species, such as *C. upsaliensis*, *C. lari,* and *C. hyointestinalis,* due to their increased association with human illness [22]. In addition, recent studies have described a new species *Candidatus C.* infans (*C. infans* from hereon), originally detected in infant stools from the Global Enteric Multicenter Study (GEMS) study[23]. This species was detected by shotgun metagenomic sequencing in 59.1% of fecal samples from diarrheal and asymptomatic children under two years of age in Iquitos, Loreto, Peru. These samples were previously tested positive for the *Campylobacter* genus but negative for *C. jejuni/coli* by PCR. *C. infans* was identified as the dominant species in breastfed infants and is associated with diarrhea in humans and non-human primates [22]. Furthermore, most studies have focused exclusively on human or animal hosts; however, the role of environmental factors, including soil and water, in *Campylobacter* transmission remains less understood.

Building on these insights, a longitudinal study was conducted from December 2020 to June 2022 aimed at assessing the prevalence, species composition, and genomic diversity of thermotolerant and non-thermotolerant *Campylobacter* spp. in infants, adults, livestock, and environmental reservoirs in the Haramaya woreda in Eastern Ethiopia. The details of the study design have been previously described [24, 25]. A high prevalence of *Campylobacter* at the genus level was observed, with 64% of stool samples from infants testing positive, with an age-dependent prevalence, as determined through TaqMan real-time PCR analysis [25]. This study seeks to elucidate the temporal colonization patterns of *Campylobacter* at the species level in infants and the potential reservoirs, [humans (siblings, mothers), livestock (cattle, sheep, goats, chickens), the environment (soil samples collected at the front and inside the home)], and household determinants contributing to their infection. Using species-specific quantitative PCR, we assessed the prevalence and load of both thermotolerant and non-thermotolerant *Campylobacter* at the species level across these various sample types and conducted risk-factor analysis using metadata obtained through extensive face-to-face interviews [26].

## Methods

### Study design, sample size, sample collection, and interviews

**Fig. 1:**
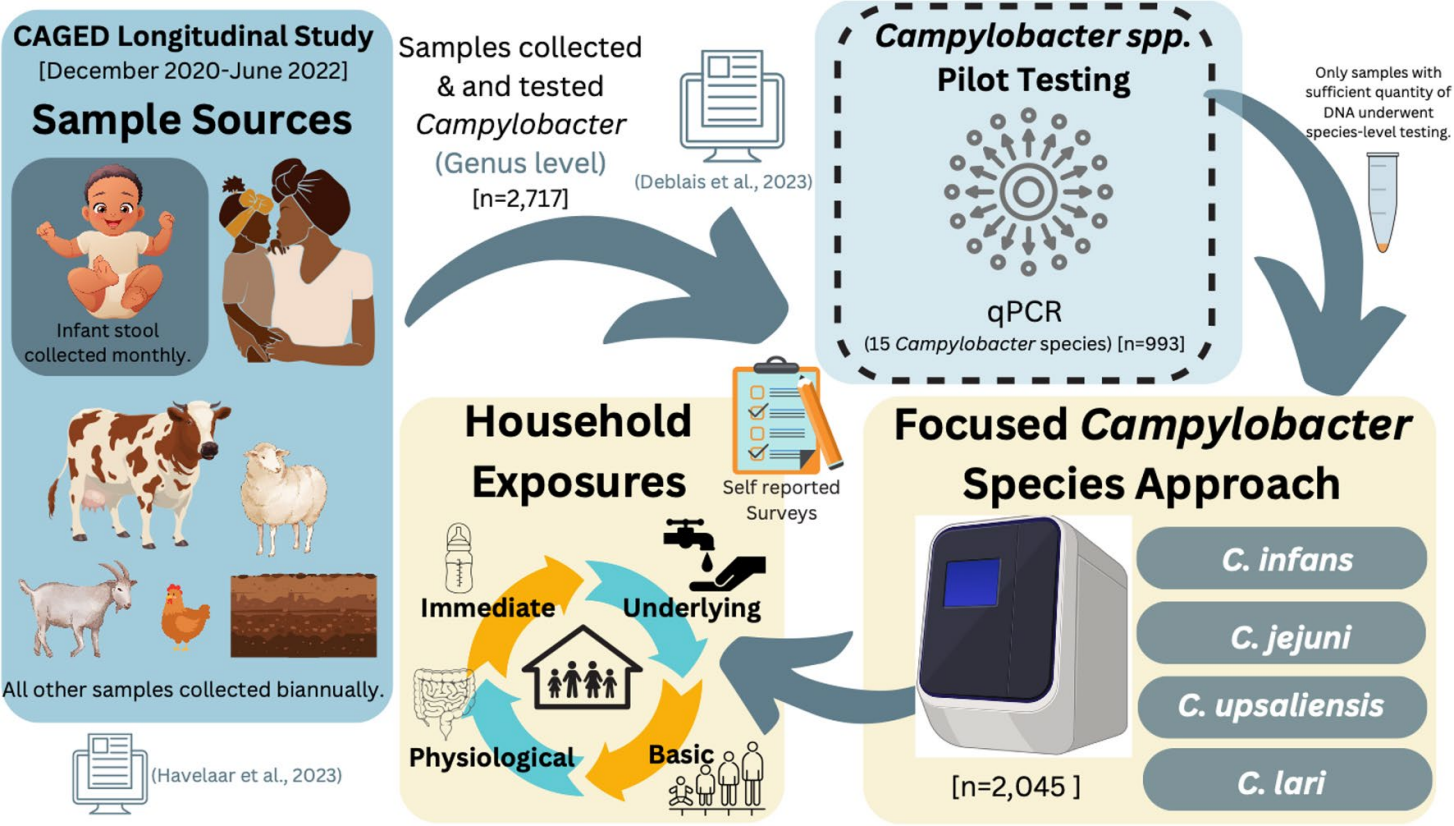
Flowchart Illustrating the Analysis Methods for the CAGED Longitudinal Cohort and *Campylobacter* Species Detection, Including the Investigation of Household Exposures Linked to Elevated *Campylobacter* Species Infections

A longitudinal study involving 106 infants was conducted. Participants were randomly selected from a birth registry in 10 kebeles (the smallest administrative unit in Ethiopia) in the Haramaya woreda, East Hararghe Zone, Oromia Region, Ethiopia (Fig. S1). Informed consent was obtained from parents in the local language (Afan Oromo). An average of 205 (minimum/maximum, 170/249) samples tested at a species level were collected per kebele. Each kebele included, on average, 11 households (minimum/maximum, 8/12), and an average of 20 (minimum/maximum, 4/28) samples per household were collected (Table S1). Infant stool samples (n = 907) were collected monthly, starting from enrollment (16–39 days after birth) until 353–375 days of age. Stools from the mother and closest-in-age sibling (n = 121 and 129) along with livestock (cattle, goat, sheep, chicken) feces (n = 640) and soil (n = 248) samples were collected twice in the first and second half year of life of the infants, (Fig. 1). Surveys were conducted during sampling to assess dietary intake and additional environmental factors. Further details on enrollment, sampling scheme, sample processing, and occurrence of *Campylobacter* at the genus level and determinants of infant colonization were described in previous publications [24–26].

### Detection of *Campylobacter* species using real-time quantitative PCR

Sample processing and DNA extraction were performed in Ethiopia using the QIAamp PowerFecal Pro kit (Qiagen, CA, USA) for human stool and livestock feces, and the DNase PowerSoil Pro DNA kit (Qiagen, Hilden, Germany) was used for soil samples as detailed in [25]. The quality and quantity of DNA were analyzed using a UV5 Nano spectrophotometer, and poor-quality samples (260/230 ratio of < 1.0; 260/280 ratio of < 1.6) were cleaned using a Zymo genomic DNA clean and concentrator kit. DNA was shipped to the United States to detect *Campylobacter* species using SYBR Green quantitative PCR. Species-specific primers targeting *hipO* and *cpn60* were used (Table 1). Each reaction mixture (25 μl final volume) contained 12.5 μl of Maxima SYBR Green/ROX qPCR Master Mix (2X), 0.5 nmol of forward and reverse primers, 50 ng of DNA, and nuclease-free water (Qiagen, Hilden, Germany). Real-time PCR runs were conducted using QuantStudio 5 (Applied Biosystems, Waltham, MA).

**Table 1:** Summary of *Campylobacter* primers and annealing temperatures

Species	Gene target	Primer sequence	Annealing (^o^C)	Product size (bp)	Product Tm (^o^C)*	References
C. jejuni*	*hipO*	GTGGTCATGGAAGTGCTCCAGAAA	58	131	78.4–79.8	[46]
		AGCTCCTATGCTTACAACTGCTGA				
*C. coli*	*cpn60*	TGACGGTAGAACTTTCAAATCC	64.6	148	78.5	[47–49]
		GCAAGTGCTTCACCTTCGATA				
C. upsaliensis*		CGTTTTGGCACACGCTATTT	64.6	110	79.5	
		CATCAACAATAGCCTCACAAGC				
C. lari*		TCTGCAAATTCAGATGAGAAAA	54.3	180	78.5	
		TTTTTCAGTATTTGTAATGAAATATGG				
*C. helveticus*		TGAAGCGATTGTTGATGAGC	64.6	151	80	
		AACGCCATCTTTTCCAACTC				
*C. fetus*		TGAGGCTGTTACAAGCGAGTTA	62.5	100	79	
		TGAGCTATCGCTATTTGCTGAA				
*C. hyointestinalis*		GGGGCAAATCCTATTGAGGT	62.5	137	79.5	
		TCGCTATTTGCAGAGATCGTAG				
*C. rectus*		AGCTATCATCGCCGAGCTAA	64.6	177	87	
		TGAATGGATTTTGCCTCCTC				
*C. showae*		GCCACTATCTCGGCAAATTC	60	125	85	
		ACGTTTAGCTCGTCGTGGAT				
*C. concisus*		GGCTCAAAAGAGATCGCTCA	64.6	158	83.5	
		CCCTCAACAACGCTTAGCTC				
*C. curvus*		CTGATAGCTGATGCGATGGA	64.6	162	83	
		CTCGACCTGCATTTTCTCG				
*C. gracilis*		AAGAGATCGCACAGGTTGCT	64.6	162	85	
		CAAACTGCATTCCCTCGACT				
*C. mucosalis*		TGCTGATGCAATGGAGAAAG	64.6	152	81	
		CCTGCATTTTCTCGGTGTTT				
*C.sputorum*		CAAAGTTGCTGAGGCAATCA	64.6	118	78	
		TGATCCAACAGCCTCATCTG				
C. infans*		ACTCAGCCGATATCATCGCC	65	119	53- 56.9	This Study
		TATCAAATCGCCAATGGCAC				

species annotated with an * asterisk represent the four tested species across all 2045 samples

### A tiered approach to *Campylobacter* species testing

Our main objective was to evaluate the diversity and prevalence of *Campylobacter* species in infants during their first 12 months of life while identifying potential reservoirs within selected households, including family members, livestock, and environmental sources. To achieve this, we implemented a tiered testing strategy comprising the following sequential steps:

#### Initial screening of 15 species

We began with a preliminary screening of 15 distinct *Campylobacter* species, including both thermotolerant and non-thermotolerant types (*C. infans, C. jejuni, C. upsaliensis, C. lari, C. concisus, C. mucosalis, C. showae, C. sputorum, C. rectus, C. gracilis, C. hyointestinalis, C. fetus, C. helveticus, C. curvus, and C. coli*) (Fig. S2). These species were selected as they have been associated with humans and based on prevalence patterns identified during the CAGED formative research [15]. The species screening was performed on a subset of samples; this approach aimed to identify species present in human stool and livestock feces.

#### Identification of seven predominant species

The initial screening identified seven *Campylobacter* species (*C. infans, C. jejuni, C. concisus, C. upsaliensis, C. mucosalis, C. showae, and C. lari)* as predominant in human stool and livestock feces. Since our primary focus was on infants, a subset of samples (a minimum of 88 infant stool samples from infants aged 8 to 367 days, with a median age of 174 days) were tested for species diversity. *C. infans*, *C. jejuni*, *C. concisus, C. upsaliensis, C. showae,* and *C. lari* were the most frequently detected species in infant stool samples (Fig. S3A). Although *C. mucosalis* was detected in 3% of samples in the pilot screening, it was positive in only one infant stool sample and thus was excluded from further testing. The remaining species were rarely detected, accounting for less than 2% of infant stool samples.

#### Focused testing of four dominant species

Guided by the pilot qPCR results and shotgun metagenomic analysis performed on a subset of samples (n = 40 per source, further details on this approach and findings will be communicated in a separate manuscript [50], we narrowed our focus to the four most prevalent and abundant species found in infant stools—*C. infans, C. jejuni, C. upsaliensis*, and *C. lari*—for comprehensive testing across all available human, livestock, and environmental samples (n = 2045).

Genomic DNAs extracted from *C. infans (*Christine Szymanski, University of Georgia, Athens, GA), *C. jejuni* (ATCC 81-176), *C. upsaliensis* (ATCC 49816), and *C.lari* (ATCC 43675) were used as positive controls. Nuclease-free water was used as a negative control. Species-specific primers were evaluated for primer sensitivity and cross-reactivity against multiple thermophilic and non-thermophilic *Campylobacter* spp. to assess their performance in targeted detection (Fig. S2). The following thermocycler conditions were used: 1 cycle at 50 °C for 2 min, 1 cycle at 95 °C for 10 min, and 40 cycles at 95 °C for 15 s, annealing temperature (Table 1) 30 s followed by 72 °C for 30 s.

Standard curves were prepared for each species using a determined concentration of DNA. The DNA concentrations in these samples were converted into genome copy numbers using the following formula: genome copies = [concentration of DNA tested (ng) × 6.0221 × 10^23^]/[average mass of 1 bp of DNA (660 g/mol) × 109] (https://Campylobacter.idtdna.com/pages/education/decoded/article/calculations-converting-from-nanograms-to-copy-number). A linear regression analysis was used to determine the relationship between the cycle threshold (CT) values and the genome copy numbers obtained for the determined concentration of DNA tested. The following equations were obtained: log_10_ *C. infans* genome copies per 50 ng DNA = [(41.04-CT)/3.74], log_10_ *C. jejuni* genome copies = [(37.62-CT)/3.75], log_10_ *C. upsaliensis* genome copies = [(39.39-CT)/3.69], log_10_ *C. lari* genome copies = [(38.32-CT)/3.94]. A CT value of < 35 was used to determine prevalence, as described in our previous publication [25].

### Integrated approach to household determinant identification

We utilized an integrated conceptual framework that merges a modified version of the United Nations International Children’s Emergency Fund (UNICEF) model with insights from a systematic review of drivers of childhood undernutrition [16]. This combined framework was used to identify potential determinants of key health outcomes, specifically *Campylobacter* infections at the species level and environmental enteric dysfunction (EED) and stunting. Details of this individual and household-level determinant analysis have been previously published [26].

The framework categorized variables into four levels: Basic, Underlying, Immediate, and Physiological causes. *Basic* causes included factors such as social/cultural conditions, economics/livelihoods, human capital, and basic demographics from the original UNICEF framework, along with variables reflecting benefits, risks, and control measures, as outlined in Chen, et al. [27]. *Underlying causes* included household food insecurity, inadequate care and feeding practices, unhealthy household environmental conditions, and inadequate health services. *Immediate causes* encompass inadequate dietary intake and diseases. *Physiological causes* focused on gut health, assessed through diarrheal symptoms and biomarkers of EED (lactulose excretion and myeloperoxidase activity). Given that prolonged infections can affect gut health, we further explored the relationship between EED status (as indicated by biomarkers for gut inflammation and permeability) and the cumulative load of *Campylobacter* infections at the species level. EED was classified using a composite indicator of lactulose excretion (%L) and fecal myeloperoxidase (MPO) levels [26]. Moderate gut permeability was defined as 0.2 < %L ≤ 0.45%, and severe permeability as %L > 0.45%. For gut inflammation, MPO thresholds were 2000 ng/ml for moderate and 3364 ng/ml for severe. EED was categorized as moderate if either %L or MPO was severe and as severe if both %L and MPO were in a severe category. Additional details on the variables included in each category are described in (Table S2).

### Statistical analysis

All statistical analyses were executed in R v4.3.1, with data securely stored in REDCap and exported as CSV files for processing and analysis [28, 29]. Differences in prevalence, co-occurrence, and abundance were assessed via Chi-square, Fisher’s exact, Wilcox, and Kruskal Wallis tests across potential exposures. Regression analyses were performed to explore the relationship between *Campylobacter* load, EED, and baseline determinants using the statistical approach described in [26]. We regressed each infant’s average *Campylobacter* load and EED status over the follow-up period on baseline determinants, excluding birth-related feeding practices. To assess the short-term effects of colostrum feeding, early breastfeeding, and prelacteal feeding, we focused on *Campylobacter* load (*C. infans, C. jejuni, C. upsaliensis*) during early infancy (7–39 days). EED was assessed in infants 12–14 months of age and regressed on baseline and time-varying determinants over the entire follow-up period. For longitudinal analysis, the average *Campylobacter* load was regressed on the average time-varying determinants within age quartiles to capture immediate effects on abundance. The same approach was used to assess the impact of *Campylobacter* burden on current enteric disease symptoms (fever and diarrhea).

Linear mixed models with individual-level random intercepts were fitted using the R package lme4 to account for repeated measures [30]. *C. infans, C. jejuni, and C. upsaliensis* load as a function of age were analyzed using generalized linear mixed models. To examine the prevalence of *Campylobacte*r species in infant stool samples, logistic regression models were fitted using generalized estimating equations. Unconditional logistic regression models were used for symptomatic and asymptomatic infections, conditioned on the presence or absence of specific *Campylobacter* species, following methods we previously established [25]. For all outcomes except enteric disease symptoms (diarrhea and fever), backward elimination was used to refine the multivariable models, starting with determinants that had adjusted p-values < 0.2 from initial screening, adjusting for sex and socio-economic status as fixed confounders.

## Results

Based on a preliminary screening of 15 *Campylobacter* species (Table 1) using a subset of samples, we identified four species most detected in infant stool: *C. infans* (36%, n = 171/475), *C. jejuni* (14%, n = 66/475), *C. upsaliensis* (10%, n = 48/475), and *C. lari* (6%, n = 30/475) (Fig. S3). Subsequently, we targeted these four species in 2,045 samples across all sources.

### Candidatus *C. infans* and *C. jejuni* predominated in infant stools

*C. infans* was detected in 41% [95% CI: 38%, 44%] of infant stools, followed by *C. jejuni* at 26% [95% CI: 23%, 28%] and *C. upsaliensis* at 13% [95% CI: 11%,15%]. *C. infans* and *C. jejuni* were detected in early infancy, as early as in the first month of life (mean 23 days old), while *C. upsaliensis* was first detected at 3 months (mean 79 days). Overall, *C. lari* prevalence was the lowest across all source types (< 2%) and was excluded from further analysis.

**Fig. 2:**
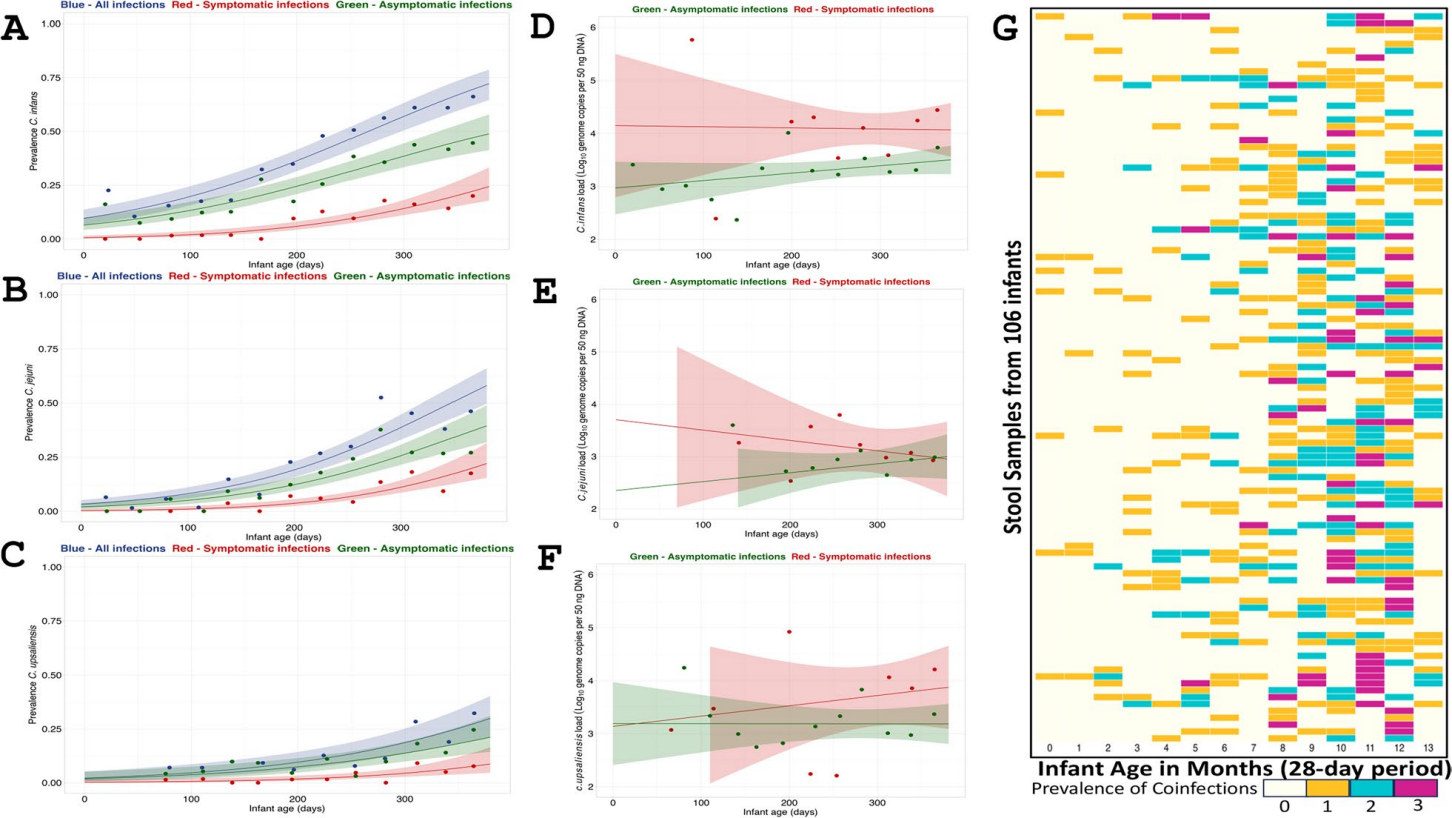
Prevalence and load of predominant *Campylobacter* spp. in infant stool samples over time. **A**–**C** Prevalence of *C. infans, C. jejuni, and C. upsaliensis* is shown by age, with points representing 4-week age groups. Lines indicate logistic regression models and shaded areas represent 95% confidence intervals. In blue are all species of infections, the red line shows symptomatic infections (presence of diarrhea) and asymptomatic infections in green. **D**–**F** *C. infans, C. jejuni, and C. upsaliensis* load in positive infant stool over time. Points are average loads by 4-week age group, lines are best-fitting linear regression models of load as a function of age as a continuous variable, and shaded areas are 95% confidence intervals. Confidence intervals are truncated between 2 and 6 log_10_ genome copies per 50 ng DNA. **G** Heatmap of *Campylobacter* spp. Coinfections (*C. infans, C. jejuni, and C. upsaliensis*) in infant stool samples, indicating prevalence by color intensity. X-axis: infant age; Y-axis: individual caged IDs. Data from 106 infants highlighting coinfections based on positive qPCR results at the genus and species level

The prevalence of *C. infans*, *C. jejuni*, and *C. upsaliensis* increased as the infants grew older, with an 0.85% increase per day in the odds of *C. infans* (*P* < 0.0001), 0.98% per day for *C. jejuni* and 0.77% per day for *C. upsaliensis* colonization (*P* < 0.0001) (Fig. 2). This increase in prevalence was associated with an increased abundance of *C. infans* and *C. jejuni* with, on average, 0.0023 log_10_ genome copies per 50 ng DNA per day and 0.0029 log_10_ genome copies per 50 ng DNA per day. The load of *C. infans* increased linearly with age (*P* = 0.027), rising from 2.75 at 10 days to 3.59 log_10_ genome copies per 50 ng DNA at 370 days. A similar trend was observed for *C. jejuni* (*P* = 0.024), which increased from 2.04 log_10_ genome copies per 50 ng DNA at 30 days to 3.05 log_10_ genome copies per 50 ng DNA at 370 days.

The co-occurrence of *Campylobacter* species in infant stool was analyzed in 28-day intervals (based on sampling scheme) to determine age-related patterns of infections. The first co-detection was observed at two months of age, and the proportion of samples with more than one species increased with age, peaking at 40% (36/78) by ten months. *C. infans*, *C. jejuni*, and *C. upsaliensis* were detected together between 4 and 13 months (Fig. 2G). The co-occurrence of *Campylobacter* species increased as infants aged, with significant associations observed at 114 to 207 days (β = 1.8123, *P* = 0.0009), 213 to 290 days (β = 2.9961, *P* < 0.0001), and 298 to 375 days (β = 3.4405, *P* < 0.0001).

### Household factors associated with *C. infans and C. jejuni* abundance in infants

Results of univariate analyses for individual- and household-level determinants (both with and without adjustment for confounders) from linear mixed modeling are summarized in (Table S2). Determinants with adjusted p values < 0.2 were included in multivariable analyses.

We observed significant associations between environmental and behavioral factors and the load of *C. infans* and *C. jejuni* in infants (Table 2). *C. infans* loads were significantly higher among infants consuming raw milk and those crawling in areas contaminated with animal droppings. Notably, female infants demonstrated consistently higher *C. infans* loads compared to males. Conversely, a lower average of *C. infans* load throughout the follow-up period was significantly associated with households with a higher sheep nighttime risk score (defined as households who confined sheep inside the home at night). Infants who put soil or animal feces in their mouths were at risk for higher *C. jejuni* loads (Table 2).

**Table 2:** Associations between *Campylobacter* species load and household determinants

Outcome	Regression coefficient	Odds ratio (95%, confidence interval)^
	Screening analysis^a^	Multivariable analysis
*C. infans Load*		
Baseline determinants^&^		
Infant sex (Female)	0.608**	1.471 (0.099, 0.674)**
Sheep nighttime location risk score(≥ 1 vs. 0)^#^	− 0.399**	0.654 (− 0.706, − 0.142)**
Disposal of infant’s stool, post-defecation	0.211	1.278 (− 0.039, 0.532)*
Handwashing after field work	− 0.304	0.769 (− 0.628, 0.105)
Longitudinal determinants		
HFIAS	0.024*	1.014 (− 0.013, 0.042)
Raw milk consumption	0.962**	2.335 (0.241, 1.455)*
Crawling where animal droppings present	0.597**	1.647 (0.066, 0.932)*
*C. jejuni* Load		
Baseline determinants^&^		
Access to drinking water	− 0.743*	0.494 (− 1.452, 0.039)*
Access to any sanitation	− 0.392*	0.757 (− 0.724, 0.165)
Mother using soap to wash hands	− 0.471 **	0.723 (− 0.744, 0.100)
Longitudinal Determinants		
HFIAS	0.040**	1.027 (− 0.009, 0.063)
Putting soil/animal feces in mouth	1.048**	2.239 (0.232, 1.381)*
Physical contact with livestock	0.694**	1.568 (− 0.055, 0.955)
*C. upsaliensis load*		
Baseline determinants^&^		
Handwashing after handling raw food	− 0.312	0.723 (− 0.729, 0.082)
Sheep daytime location risk score(≥ 1 vs. 0)^#^	− −0.546**	0.606 (− 1.028, 0.026)*
Collection of livestock waste	− 0.544	0.653 (− 1.067, 0.215)
Longitudinal determinants		
Achieved MDD	0.363	1.437 (− 0.184, 0.910)

*HFIAS* household food insecurity access score

^**^Represents p ≤ 0.05. ^*^ Represents 0.05 ≤ p < 0.1

^^^Backward elimination suggested the final model was the single-exposure model. Further details on the screening results can be found in Table S2

^^^Adjusted for socio-economic status at baseline and sex

^#^ ≥ 1: Households kept the livestock species inside the house confined or unconfined; 0: Households did not have or keep the livestock species outside the house

^&^The average *Campylobacter* load in an age quartile was regressed on each determinant’s concurrent proportion/mean, adjusting for age quartile, sex, and socioeconomic status at baseline. Linear-mixed models with individual-level random intercepts were used to estimate the coefficients

^a^Screening Analysis represents findings from univariate analysis in which determinants with adjusted p-values < 0.2 were included in the multivariable analysis

#### Enteric disease symptoms

Most stool samples positive for *Campylobacter* were collected from asymptomatic infants (n = 682/907). However, *C. infans* colonization was significantly associated with diarrheal episodes (OR = 2.02, β = 0.70, P = 0.000105, [95%CI: 35%, 100%]) and fever (OR = 1.62, β = 0.48, P = 0.006, [95%CI: 14%, 83%]). Among the 346 stool samples positive for *C. infans*, 25% (85/346) were from infants experiencing diarrhea, and 24% (83/346) had a fever. Similarly, *C. jejuni* showed a significant association with diarrheal episodes (OR = 2.29, β = 0.83, P < 0.0001[95%CI: 46%, 100%]) and fever (OR = 2.53, β = 0.92, P < 0.0001, [95%CI: 56%,100%]), with 28% (61/214) of the 214 positive stool samples collected during episodes of diarrhea and 32% (68/214) with fever. Additionally, in symptomatic infants, we observed a daily increase of 0.54% in the prevalence of *C. infans* and 0.53% in the prevalence of *C. jejuni* (P = 0.0008 and P = 0.024, respectively) (Fig. 2A–F). When exploring the impact of *Campylobacter* abundance and enteric disease symptoms, we found higher *C. infans* loads were associated with diarrhea and fever (Table 3). However, fever was not statistically significant after adjustment for confounders. No statistical associations were found between enteric symptoms and the abundance of either *C. jejuni* or *C. upsaliensis* or the prevalence of *C. upsaliensis*.

**Table 3:** Associations between *Campylobacter* species load and enteric disease symptoms

Odds ratio (95%, Confidence Interval)^^^						
Outcome	*C. infans load*		*C. jejuni load*		*C. upsaliensis load*	
	Unadjusted	Adjusted	Unadjusted	Adjusted	Unadjusted	Adjusted
Diarrhea	1.345 (0.056, 0.539)*	1.384 (0.061, 0.589)*	1.071 (− 0.093, 0.231)	1.071 (− 0.12, 0.259)	0.95 (− 0.245, 0.144)	0.952 (− 0.251, 0.153)
Fever	1.039 (0.014, 0.063)*	1.019 (− 0.007, 0.044)	1.049 (0.033, 0.063)	1.039 (0.021, 0.057)	1.013 (− 0.007, 0.033)	1.005 (− 0.015, 0.025)

Using a linear mixed model with individual-level random intercept, the proportion of each symptom’s occurrence was regressed as the outcome on the concurrent average *Campylobacter* load using crude and adjusted models (adjusting for age quartile and infant sex at baseline)

^*^p < 0.05

#### EED

At 1 year of age, EED was identified in 54 out of 101 infants, with 56% [95%CI: 42%, 69%] classified as moderate and 44.4% [95%CI: 31%, 59%] as severe. Intestinal inflammation, based on MPO levels, was present in all EED cases, with severe inflammation observed in 46% [95%CI: 33%, 60%]. Elevated lactulose levels indicated intestinal permeability in 52 out of 99 infants, with severe permeability detected in 52% [95%CI: 38%, 66%]. Despite the high prevalence of EED, no significant associations were found between the bacterial load of *C. infans, C. jejuni*, or *C. upsaliensis* and EED. Similarly, there were no significant correlations between bacterial load and individual biomarkers of gut inflammation and permeability (Table 4).

**Table 4:** Associations between *Campylobacter* species load and gut health indicators

	Odds ratio (95%, Confidence interval)					
Outcome	*C. infans *load		*C. jejuni *load		*C. upsaliensis *load	
	Unadjusted	Adjusted ^^^	Unadjusted	Adjusted^^^	Unadjusted	Adjusted^^^
Lactulose percentage	1.038 (0.627, 1.719)	1.016 (0.603, 1.713)	1.024 (0.672, 1.560)	1.031 (0.674, 1.579)	0.992 (0.692, 1.422)	0.998 (0.695, 1.433)
Fecal myeloperoxidase	1.038 (0.626, 1.721)	0.984 (0.580, 1.669)	0.901 (0.591, 1.373)	0.910 (0.591, 1.401)	0.963 (0.671, 1.381)	0.980 (0.680, 1.413)
Environmental enteric dysfunction	1.386 (0.826, 2.324)	1.384 (0.808, 2.372)	1.025 (0.673, 1.563)	1.030 (0.669, 1.585)	1.020 (0.710, 1.463)	1.035 (0.718, 1.492)

^^^Adjusted for infant sex and age at EED sampling

### *C. infans* and *C. jejuni* are common in Ethiopian household environments

#### Family members

*C. infans* was frequently detected in stools from both siblings (under 5 years of age) and mothers, with a prevalence of 56% [95% CI: 48%, 65%] in siblings and 45% [95% CI: 36%, 54%] in mothers. The second most common species, *C. jejuni*, showed a higher prevalence in siblings at 24% [95% CI: 16%, 31%] compared to mothers at 9% [95% CI: 3%, 14%] (*P* = 0.07). *C. upsaliensis* was detected in 9% of all human stool samples (23/250), with siblings showing a higher prevalence at 12% [95% CI: 6%, 18%] versus 6% in mothers [95% CI: 1%, 9%].

**Fig. 3:**
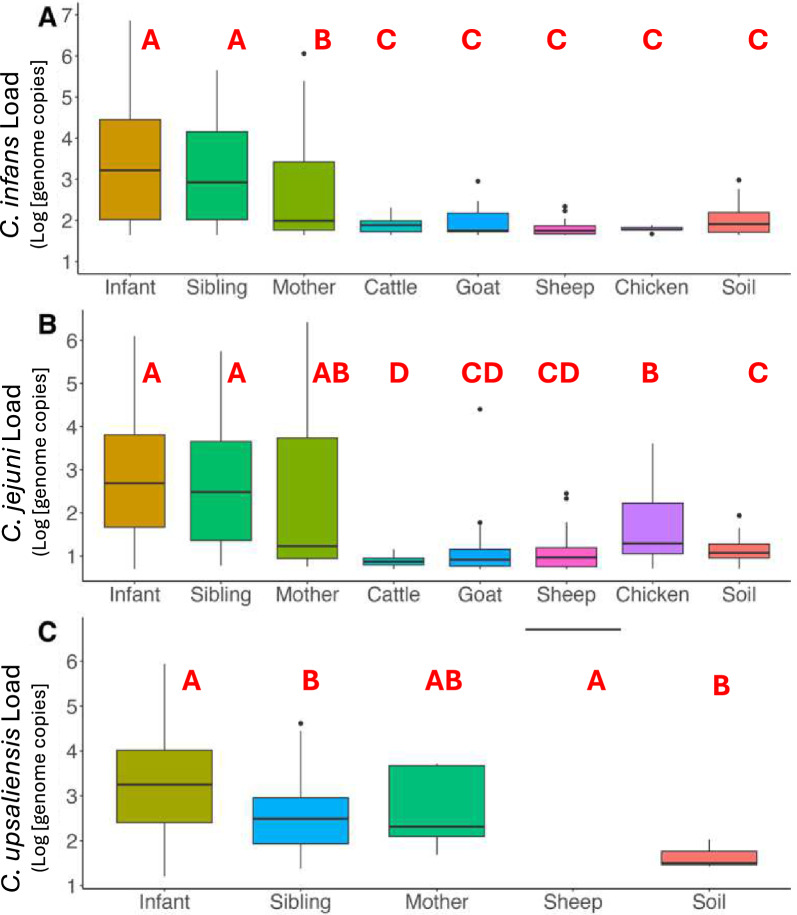
Abundance of *C. infans (A)*, *C. jejuni (B),* and *C. upsaliensis (C)* in 106 households. The boxplot shows bacterial load in positive samples (log[genome copies per 50 ng of DNA]). Letters **A**–**D** indicate statistical differences (*P* < 0.05) based on Kruskal Wallis and post hoc Dunn’s test

While the abundance of *C. infans* was overall high in humans, siblings had the highest average load after infants, with an average of 3.15 log_10_ genome copies per 50 ng DNA [95% CI: 2.86, 3.43], compared to mothers who had an average load of 2.67 [95% CI: 2.34, 2.99] (*P* = 0.0118) in positive *C. infans* samples. In contrast, no significant differences were observed in the loads of *C. jejuni* or *C. upsaliensis* between siblings and mothers. For *C. jejuni*, the average load was 2.67 [95% CI: 2.17, 3.15] in siblings and 2.39 [95% CI: 1.16, 3.62] in mothers. For *C. upsaliensis*, the average load was 2.59 [95% CI: 2.13, 3.06] in siblings and 2.75 [95% CI: 2.08, 2.75] in mothers.

Given the high prevalence of *Campylobacter* in humans, we examined the likelihood of multiple species co-occurring within the same sample. Like infants, siblings exhibited a high co-occurrence of multiple *Campylobacter* species that were significantly different from mothers (OR = 2.12, 95% CI: 1.07–4.35, β = 0.752, *P* = 0.033).

#### Zoonotic sources

Among the livestock studied in the 106 households, *C. jejuni* prevalence was highest in chickens at 38% [95% CI: 28%, 47%] and small ruminants, including goats, at 27% [95% CI: 21%, 34%] and sheep at 21% [95%CI: 15%, 28%] (Fig. 3). Among livestock species, *C. infans* was most frequently detected in sheep at 13% [95% CI: 8%, 19%], followed by goats (10%, [95%CI: 6%, 15%] and cattle (9%, [95% CI: 6%, 15%] with chickens having the lowest occurrence at 4.8% [95%CI: 2%, 11%]. However, most of these samples had high CT values (around 35), suggesting low abundance of *C. infans* in these reservoirs (Fig. S4). The co-occurrence of multiple species was low across all zoonotic sources, at < 5% [95% CI: 5%, 13%].

In samples positive for *C. jejuni*, the load ranged from 0.7 to 4.4 log_10_ genome copies, with average of 1.17 [95% CI: 1.07, 1.26]. Chicken feces had higher *C. jejuni* loads (1.62 [95% CI: 1.37,186]) compared to cattle (0.87 [95% CI: 0.83,0.92] log_10_ genome copies per 50 ng of DNA (*P* = 0.0027, Fig. 3). In *C. infans* positive samples, the load ranged between 1.6 and 2.9 log_10_ genome copies. Goats had the highest average load of *C. infans* across all livestock, 1.95 [95% CI: 1.78, 2.12]; however, no statistical differences were observed in abundance between livestock species. *C. upsaliensis* was detected in one sheep sample with a load of 6.7 log_10_ genome copies per 50 ng of DNA.

#### Household soil samples

In soil samples, *C. infans* was the most prevalent at 33% [95% CI: 27%, 38%], followed by *C. jejuni* at 19% [95% CI: 14%, 23%], while *C. upsaliensis* was rare, constituting < 2% [95% CI: 0%, 3%]. Co-occurrence was observed in approximately 10% of soil samples [95%CI: 5%, 13%], in which samples collected from inside the home had a greater prevalence (17%, [95%CI: 5%, 29%]). The average load of *C. infans* and *C. jejuni* in positive soil samples was 2.04 [95% CI: 1.96, 2.11] and 1.18 [95% CI: 1.08, 1.29] log_10_ genome copies per 50 ng of DNA.

### Comparative analysis of *Campylobacter* genus and species identification

Cross-tabulation was conducted to evaluate the concordance between genus-level *Campylobacter* detection [25] and the detection of specific *Campylobacter* species in this study. Among 2,045 samples from humans, livestock, and soil, 1622 tested positive at the genus level. Of these, 865 were also positive for one or more of the tested species, while 340 samples tested negative for both genus and species-level tests. Interestingly, 83 samples tested positive for one or more species despite being negative at the genus level.

Among infant stool samples, 594 tested positive at the genus level, of which 444 were also positive for one or more of the tested *Campylobacter* species. While 264 samples tested negative for both genus and species-level PCRs, and 48 samples tested positive for one or more species but were negative at the genus level (Table 5). A significant association was observed between genus- and species-level positivity, with more likely to detect one or more species in genus-level positive samples compared to genus-negative samples (P < 0.0001). These findings suggest that the three species detected in our study account for a substantial proportion of the *Campylobacter* genus identified in these samples.

**Table 5:** Concordance of Genus and Species-specific *Campylobacter* Detection in Infant Stool

	Genus (−)	Genus (+)
All sources (n = 2045)		
One species	18% (75/423)	37% (597/1622
Multiple species	2% (8/423)	17% (268/1622)
No species	80% (340/423)	46% (757/1622)
Infant stool (n = 907)		
One species	14% (43/313)	43% (254/594)
Multiple species	1% (5/313)	32% (190/594)
No species	85% (265/313)	25% (150/594)

This table presents a comparative analysis of *Campylobacter* species detection with genus-level test results across different sample sources. Of the 2045 collected samples, 1622 tested positive for *Campylobacter* at the genus level. Within the subset of infant stool samples (n = 907), 594 were confirmed positive for the *Campylobacter* genus

## Discussion

In low- and middle-income countries, diarrheal diseases remain a leading cause of mortality in children under 5 years of age, with *Campylobacter* species emerging as significant pathogens associated with both acute illness and chronic health consequences, including malnutrition, stunting, and cognitive deficits [8, 27, 31]. The CAGED longitudinal study found *Campylobacter* is highly prevalent in rural households in e﻿astern Ethiopia [21]. Here, we identified a high prevalence of *C. infans* and *C. jejuni* among infants residing in Haramaya, Ethiopia, using quantitative PCR (qPCR). Over 20% of infants were colonized with *C. infans* within the first month of life, and the prevalence plateaued at 61% by 11–12 months. In contrast, *C. jejuni* colonization was observed in 6% of infants during the first month, peaking at 53% by 10 months. *C. upsaliensis* colonization was observed later, emerging after the first two months of life, and remained relatively low (6–12%) throughout most of the first year. However, a notable increase to 28% at 11 months suggests that *C. upsaliensis* colonization may occur later in infancy. These findings align with research efforts to understand *Campylobacter* acquisition and clearance dynamics using the MAL-ED longitudinal data, which also observed increased *Campylobacter* acquisition rates in infants across multiple low- and middle-income countries during the first year of life [31]. Both *C. infans* and *C. jejuni* abundances showed positive correlations with infant age, ranging between 2 to 3 log_10_ genome copies per 50 ng DNA, which is concerning as *Campylobacter* is highly infectious compared to other gastrointestinal pathogens, given that *C. jejuni* has a minimum infectious dose of 500 to 800 organisms [32, 33]. In addition, the co-occurrence of *Campylobacter* species increased as infants grew older, with infants between 298 and 375 days old having 31 times higher odds of co-occurrence detection. Overall, the prevalence and loads of infants at approximately one year of age were higher than those in siblings, which in turn were higher than in mothers. These differences are likely due to the development of immune systems and differences in environmental exposure through behaviors such as crawling and play and different hygiene practices. The development of adaptive immunity may play a crucial role in age-related susceptibility to *Campylobacter* [31]. Acute infections trigger the production of specific immunoglobulins (IgG, IgM, and IgA) in serum and intestinal secretions, with IgA and IgM antibodies particularly elevated during acute phases [34–37]. This acquired immunity strengthens with repeated exposures throughout life [38], likely explaining the lower infection and co-occurrence rates observed in mothers compared to their children.

In our study, many *Campylobacter*-positive samples belonged to asymptomatic infants; however, infants colonized with *C. infans* had 2.0 times higher odds of experiencing enteric symptoms, particularly diarrhea, which was linked to higher *C. infans* load. These findings contrast with recent observations from other LMICs, particularly from Peru, where researchers reported low *C. infans* prevalence in infants under two years of age and found no association between *C. infans* colonization and diarrheal symptoms [39, 40]. While the Peru cohort was small, this discrepancy highlights the importance of conducting region-specific epidemiological studies. It suggests that the role *C. infans* in infant health may be more complex than initially understood, warranting further investigation into strain-specific characteristics and local determinants of pathogenicity. Additionally, we found that infants colonized with *C. jejuni* had 2.3 times higher odds of experiencing diarrhea, aligning with findings from other LMIC-oriented studies, including the GEMS and MAL-ED studies, which identified *C. jejuni* as a leading cause of bacterial diarrhea in LMICs during early childhood [7, 41, 42]. Despite the high occurrence of *Campylobacter* infection and EED, with more than half of infants having EED by one year of life, no association was found between *C. infans, C. jejuni*, or *C. upsaliensis* load and EED in this population. These results align with previous work assessing the relationship between the overall *Campylobacter* load and gut function parameters [26] and is related to a small sample size in our study. Importantly, we observed similar loads of *Campylobacter* at the genus level and the three dominant species in samples from diarrheal and non-diarrheal infants, supporting the finding from the MAL-ED study that asymptomatic infants are also at risk of developing chronic gut inflammation and, consequently, EED and stunting [43]. It is important to note, however, that the associations observed between *Campylobacter* loads, symptoms, and environmental exposures in our study are correlational. While these findings suggest potential links, the study design does not allow us to infer causality, and further research is necessary to determine whether these environmental factors directly influence infection rates.

Several household determinants were identified to impact *Campylobacter* species abundance. Household dietary practices significantly influenced *Campylobacter* abundance, with raw milk consumption strongly associated with higher *C. infans* loads. This association was also observed at the genus level, suggesting that introducing complementary foods, particularly animal-source foods, may be a critical source for microbial colonization [16, 27]. Infant sex also influenced *Campylobacter* colonization, as females had a higher average load of *C. infans*, suggesting potential gender-based differences in susceptibility and/or exposure. Additionally, crawling in areas with animal droppings was linked to increased *C. infans* load. While *C. infans* was not prevalent among the four livestock species tested in this study, its detection in surface soil samples around the household underscores the potential for complex environmental exposure pathways contributing to elevated *C. infans* levels in infants. While sheep demonstrated the highest *C. infans* prevalence among tested livestock species, closer nighttime proximity to sheep was associated with lower infant *C. infans* loads, matching findings from genus-level testing. Similarly, keeping sheep confined inside the home was associated with a reduced abundance of *C. upsaliensis*. This pattern may suggest that though sheep are reservoirs of *Campylobacter*, the relationship between animal proximity and human colonization is complex and requires further investigation. Given that these findings originate from rural households, where there is frequent close contact with livestock and environmental exposures, it would be beneficial to conduct similar studies in urban settings. Urban environments differ significantly in terms of infrastructure, sanitation, and patterns of animal-to-human contact, which can influence the prevalence and transmission pathways of *Campylobacter* species. Lastly, we found that infants who put soil or animal feces in their mouths were at risk for higher *C. jejuni* loads, a species predominant in zoonotic reservoirs. These findings emphasize the need for targeted interventions to address the complex interplay of environmental, dietary, and behavioral factors in *Campylobacter* transmission. Such interventions are urgently required to reduce infection rates and mitigate the burden of diarrheal diseases in resource-limited settings.

Building on our formative research, this longitudinal study provides deeper insights into the socio-demographic and exposure dynamics within the Haramaya woreda and may also be relevant for comparable smallholder environments. Our species-specific testing across human, livestock, and environmental samples revealed that three targeted *Campylobacter* species accounted for 54% of genus-level detections, suggesting the presence of additional, untested *Campylobacter* species and undetected species using the current method in these ecological niches. Indeed, shotgun metagenomic testing identified 21 dominant *Campylobacter* species across 280 samples (infants, mothers, siblings, cattle, goats, sheep, and chickens), revealing a diverse *Campylobacter* community in which many different species were found in ruminants that do not seem to be transmitted to humans. In infant stools, however, *C. infans, C. jejuni*, and *C. upsaliensis* collectively accounted for 75% of the *Campylobacter* genus-level signals, establishing these species as the predominant colonizers of the infant gut. This finding aligned with metagenomic data, where *C. jejuni, C. infans*, and *C. upsaliensis* were frequently detected alongside *C. concisus* [50]. In addition, 83 samples tested positive for one or more species, while the test at the genus level was negative. TaqMan was used for genus testing, whereas species testing was conducted with SYBR Green, and these observations may be partly explained by the different sensitivities of the two PCR methods. Further emphasizing these limitations, Parker et al. (2022) demonstrated that even validated primers for *C. jejuni* can yield false negatives. They found that a significant percentage of stool samples from children in Peru tested negative for *C. jejuni* using qPCR. Yet, the species was detected via whole-genome shotgun metagenomic sequencing performed on the same extracts. While we utilized validated primers for species testing, we acknowledge certain limitations in our approach. We observed some cross-reactivity in our primers, particularly for *C. jejuni* (with *C. fetus, C. lari*, and *C. showae*), *C. upsaliensis* (with *C. helveticus*), and *C. lari* (with *C. helveticus* and *C. upsaliensis*). *C. infans* primers showed minimal cross-reactivity with *C. concisus* at a high CT value, indicating low-level amplification. Future studies could benefit from a combined approach using both qPCR and metagenomic sequencing to address these limitations. While qPCR offers higher sensitivity and is more suitable for the absolute quantification of target genes, metagenomic sequencing provides broader coverage and an unbiased overview of microbial species within a sample [44]. This complementary approach would leverage the strengths of both methods: the high sensitivity and quantification capabilities of qPCR and the comprehensive detection and novel species identification potential of metagenomics [45]. By integrating these techniques, researchers can achieve a more robust surveillance strategy, gaining a deeper understanding of the microbial landscape while minimizing diagnostic gaps and potential biases introduced by primer-based methods.

This study contributes to the growing body of evidence elucidating the prevalence and diversity of *Campylobacter* species in human and animal populations. Our investigation revealed distinct distribution patterns: *C. infans* showed a high occurrence in human stool (cumulatively, 43% prevalence, p < 0.001 based on the chi-square test), which increased during the first year of life, and in surface soil samples, while *C. jejuni* demonstrated substantial presence across environmental and zoonotic sources, particularly in livestock. Consistent with previous research, our findings reveal that human stools and livestock feces frequently harbor multiple *Campylobacter* species. Our species-specific quantitative PCR results, further validated through shotgun metagenomics analyses in a subset of samples (n = 280, 40 per source), not only confirmed the presence of targeted *Campylobacter* species but also suggested the existence of additional species, particularly in ruminants [50]. These complementary approaches strengthen our confidence in the observed prevalence patterns and underscore the value of refined detection methodologies.

## Conclusions

In this study, we investigated the prevalence and dynamics of *Campylobacter* species colonization among 106 households residing in Haramaya, Ethiopia, using species-specific quantitative PCR. We identified *C. infans, C. jejuni,* and *C. upsaliensis* as the predominant colonizers of the infant gut, with notable age-dependent patterns and associations with enteric symptoms. Notably, *C. infans* and *C. jejuni* were linked to higher odds of diarrhea and fever, highlighting their clinical significance. Our findings support the recognition of Candidatus C. infans as a novel species with a distinct ecology. In this setting, *C. infans* was more prevalent in humans than *C. jejuni*, suggesting primarily human-to-human transmission. The low occurrence in livestock implies potential reverse zoonotic transmission or passage of this bacteria through the animal gut after exposure from a highly contaminated environment. The co-presence of anthroponotic *C. infans* and zoonotic *C. jejuni* and *C. upsaliensis* suggests that control methods in low-resource settings should address both transmission cycles. The identified risk factors and complex interplay of environmental, dietary, and behavioral factors offer potential targets for interventions to reduce *Campylobacter* colonization and associated health impacts.

## Supplementary Information

Supplementary file 1.

## Data Availability

Deidentified individual participant data will be available through Dataverse (https://dataverse.org/) after December 31, 2024. No datasets were generated or analysed during the current study.
