# Supplementary material for: Determinants of Campylobacter species diversity in infants and association with family members, livestock, and household environments in rural Eastern Ethiopia
Source: Gut Pathog. 2025 Jul 5;17:51. doi: 10.1186/s13099-025-00725-0 (PMC12228300; doi:10.1186/s13099-025-00725-0)
Supplement: Supplementary file 1 [file 13099_2025_725_MOESM1_ESM.pdf]

Title: Determinants of *Campylobacter* species diversity in infants and association with family members, livestock, and household environments in rural Eastern Ethiopia.

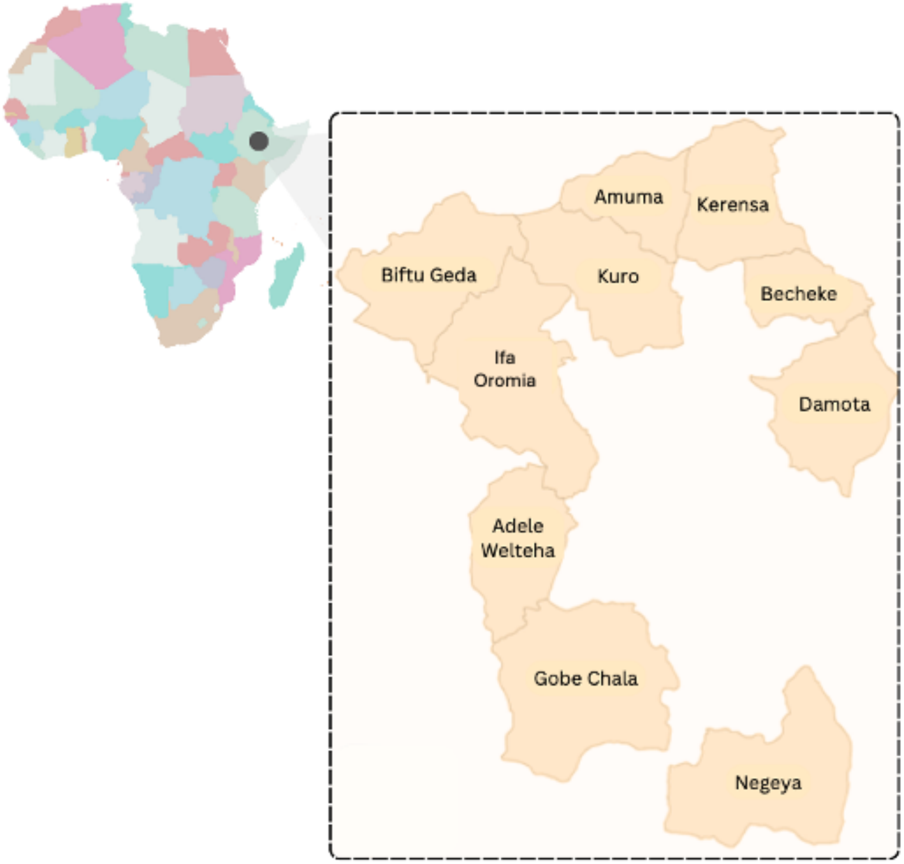


**Fig. S1. Geographic locations of the households selected for the CAGED longitudinal study.** A total of 106 households located within ten kebeles from the Haramaya woreda in eastern Ethiopia were selected and followed for one year. Additional details about the population size and sample types for each kebele are included in Table S1.

Title: Determinants of *Campylobacter* species diversity in infants and association with family members, livestock, and household environments in rural Eastern Ethiopia.

| DNA Used (50ng) | Species-specific primers | | | | | | | | | | | | | | |
|---|---|---|---|---|---|---|---|---|---|---|---|---|---|---|---|
| | *C. fetus* | *C. helveticus* | *C. hyointestinalis* | *C. lari* | *C. sputorum* | *C. upsaliensis* | *C. concisus* | *C. rectus* | *C. showae* | *C. infans* | *C. jejuni* | *C. gracillis* | *C. curvus* | *C. mucosalis* | *C. coli* |
| *C. coli* ATCC33559 | 42 | 40 | 36 | 42 | 41 | 37 | 42 | 42 | 42 | 42 | | 42 | 42 | | 15 |
| *C. fetus* #33293 | 13 | 37 | 37 | 33 | 36 | 35 | 42 | 42 | 38 | 42 | 35 | 42 | 42 | 42 | |
| *C. helveticus* #51209 | 33 | 14 | 34 | 34 | 36 | 27 | 42 | 41 | 39 | 40 | 42 | 42 | 42 | 42 | |
| *C. hyointestinalis* #35217 | 37 | 36 | 13 | 42 | 37 | 36 | 42 | 39 | 39 | 42 | 42 | 42 | 42 | 42 | |
| *C. jejuni* 81-176 | 42 | 42 | 42 | 42 | 42 | 42 | 42 | 42 | 42 | 42 | 13 | 42 | 42 | 42 | |
| *C. lari* #43675 | 36 | 38 | 36 | 13 | 36 | 37 | 39 | 42 | 42 | 42 | 33 | 42 | 42 | 42 | |
| *C. sputorum* #49916 | 38 | 38 | 37 | 36 | 14 | 37 | 38 | 42 | 42 | 42 | 42 | 42 | 42 | 42 | |
| *C. upsaliensis* #49816 | 34 | 18 | 34 | 28 | 38 | 12 | 41 | 37 | 42 | 42 | 34 | 42 | 42 | 42 | |
| *C. concisus* #51561 | 42 | 42 | 42 | 42 | 41 | 42 | 18 | 42 | 42 | 32 | 42 | 42 | 42 | 42 | |
| *C. rectus* #33238 | 42 | 42 | 42 | 42 | 38 | 37 | 38 | 24 | 38 | 42 | | 42 | 42 | 42 | |
| *C. showae* #51146 | 42 | 40 | 38 | 38 | 38 | 42 | 40 | 38 | 20 | 36 | 35 | 42 | 42 | 42 | |
| *C. hominus* #15827 | 42 | 42 | 41 | 42 | 42 | 42 | 42 | 42 | 42 | | | | | | |
| *C. infans* | | | | | | 42 | | | | 14.3 | 42 | 42 | 42 | 42 | |

**Fig. S2. *Species-specific primer specificity among Campylobacter species.*** A total of 15 *Campylobacter* species-specific primers were tested against several thermophilic and non-thermophilic species, using specific *Campylobacter* primers targeting *hipO* or the *cpn60* gene (Chaban et al, 2009)—50 ng of DNA per reaction with three replicates per species tested. Blank cells reflect species that were not tested. DNA was unavailable for *C. gracillis*, *C. curvus*, and *C. mucosalis*. The cell color corresponds to the Ct value (dark purple to blue = low to high). #refers to American Type Culture Collection (ATCC) number.

Title: Determinants of *Campylobacter* species diversity in infants and association with family members, livestock, and household environments in rural Eastern Ethiopia.

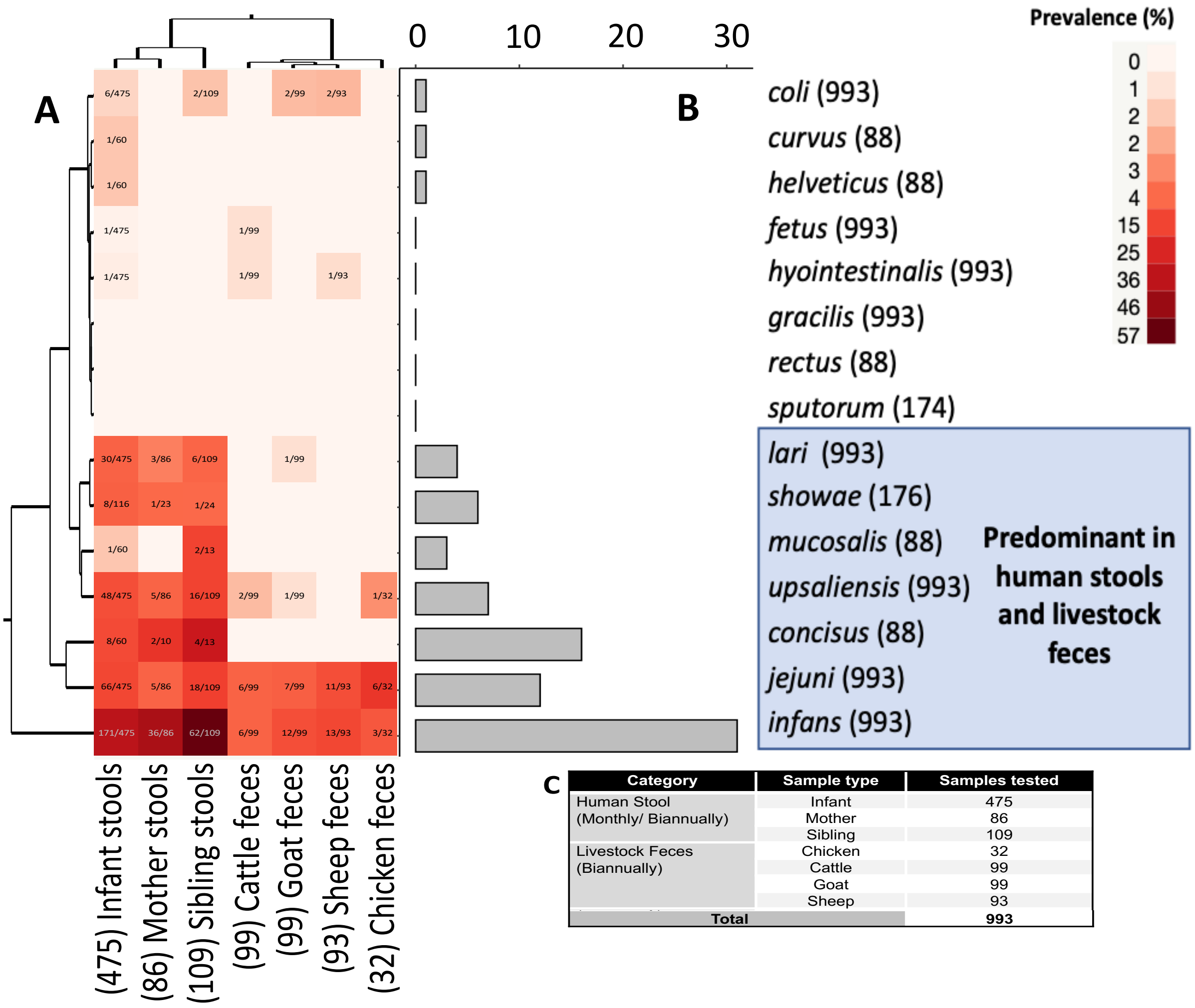


| Category | Sample type | Samples tested |
|---|---|---|
| Human Stool (Monthly/ Biannually) | Infant | 475 |
| | Mother | 86 |
| | Sibling | 109 |
| Livestock Feces (Biannually) | Chicken | 32 |
| | Cattle | 99 |
| | Goat | 99 |
| | Sheep | 93 |
| **Total** | | **993** |

**Fig. S3. Findings tiered approach to *Campylobacter* species testing.** (**A**) *Campylobacter* species prevalence in human stools and livestock feces pilot test using two-way clustering plot**.** The color of the cells is proportional to the prevalence values (positive samples / total population tested). Numbers in parentheses indicate the overall population size tested. Empty cells: species not detected. (**B**) Total Prevalence % of *Campylobacter* spp. Detected. Prevalence values were determined by calculating (positive samples / total population tested). The total population tested per species varied based on the number of samples tested. Ct value cut-off of 35 was used to detect *Campylobacter* in the field samples across all species. (**C**) Sampling summary of *Campylobacter* species pilot test**.**

Title: Determinants of *Campylobacter* species diversity in infants and association with family members, livestock, and household environments in rural Eastern Ethiopia.

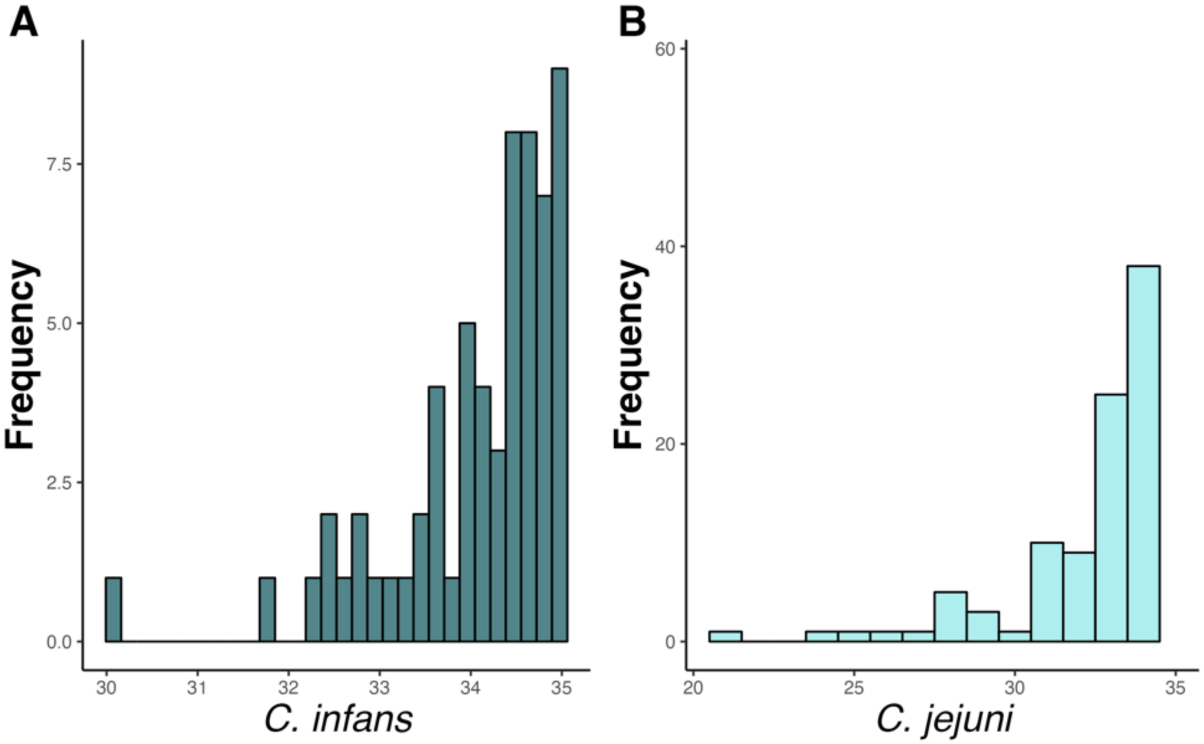


**Fig. S4. CT Distribution of Livestock samples positive for (A) *C .infans* and (B) *C. jejuni*.**

Title: Determinants of *Campylobacter* species diversity in infants and association with family members, livestock, and household environments in rural Eastern Ethiopia.

**Table S1. Sample size by source for *Campylobacter* spp. Testing using real-time quantitative PCR per kebele.**

| KEBELES (N=10) | HOUSEHOLDS SELECTED | SAMPLES TESTED | ENVIRONMENT | | | | | Human | | |
|---|---|---|---|---|---|---|---|---|---|---|
| | | | Soil | Cattle | Goat | Chicken | Sheep | Infant | Mother | Sibling |
| NEGEYA | 12 | 236 | 32 | 22 | 22 | 15 | 20 | 102 | 11 | 12 |
| DAMOTA | 12 | 232 | 26 | 23 | 21 | 13 | 17 | 99 | 17 | 16 |
| GOBE CHALLA | 9 | 177 | 29 | 14 | 17 | 8 | 16 | 70 | 10 | 13 |
| BIFTU GEDA | 11 | 217 | 22 | 18 | 17 | 10 | 18 | 102 | 13 | 17 |
| IFA OROMIA | 10 | 192 | 28 | 19 | 18 | 10 | 19 | 79 | 9 | 10 |
| AMUMA | 11 | 205 | 32 | 17 | 16 | 10 | 18 | 94 | 7 | 11 |
| KURO | 11 | 186 | 32 | 18 | 16 | 10 | 14 | 78 | 10 | 8 |
| ADELE WALTA | 10 | 181 | 6 | 17 | 16 | 9 | 16 | 87 | 16 | 14 |
| QERENSA DEREBA | 12 | 249 | 20 | 21 | 22 | 14 | 23 | 119 | 16 | 14 |
| BACHAQE | 8 | 170 | 21 | 14 | 14 | 5 | 13 | 77 | 12 | 14 |
| **TOTAL** | **106** | **2045** | **248** | **183** | **179** | **104** | **174** | **907** | **121** | **129** |

Title: Determinants of *Campylobacter* species diversity in infants and association with family members, livestock, and household environments in rural Eastern Ethiopia.

## Table S2. Determinants of *Campylobacter* species load.

| Domain | | Determinant[$] | *C.infans* load *(P-value)* | | *C.jejuni* load *(P-value)* | | *C. upsaliensis* load *(P-value)* | |
|---|---|---|---|---|---|---|---|---|
| | | | Unadjusted | Adjusted[^] | Unadjusted | Adjusted[^] | Unadjusted | Adjusted[^] |
| Immediate Causes | Inadequate Dietary intake | **Time-varying determinants** | | | | | | |
| | | Achieved MDD[#] | 0.094 (0.688) | -0.136 (0.555) | 0.280 (0.409) | 0.098 (0.769) | 0.696 (0.014)* | 0.363 (0.195) |
| | Disease | **Baseline determinants** | | | | | | |
| | | WAZ[#] at enrollment | -0.077 (0.249) | -0.061 (0.358) | -0.059 (0.472) | -0.068 (0.409) | -0.140 (0.127) | -0.145 (0.118) |
| | | Underweight at enrollment | 0.330 (0.170) | 0.280 (0.236) | 0.032 (0.913) | 0.063 (0.831) | 0.212 (0.521) | 0.237 (0.479) |
| Underlying Causes | Household food security | **Time-varying determinants** | | | | | | |
| | | HFIAS[#] | 0.049 (0.001)* | 0.024 (0.087) | 0.068 (0.000)* | 0.040 (0.024)* | 0.024 (0.178) | 0.010 (0.584) |
| | | Food Secure (i.e., HFIAS = 1) | -0.634 (0.000)* | -0.171 (0.313) | -0.968 (0.000)* | -0.259 (0.246) | -0.296 (0.149) | 0.120 (0.557) |
| | Inadequate care and feeding practices | **Baseline determinants** | | | | | | |
| | | Colostrum feeding | -0.281 (0.422) | -0.526 (0.253) | 4.108 (0.9969) | 4.043 (0.997) | 5.978 (0.997) | 5.885 (0.997) |
| | | Pre-lacteal feeding | -0.092 (0.713) | -0.13 (0.615) | -4.620 (0.994) | -4.536 (0.994) | -6.724 (0.994) | -6.602 (0.994) |
| | | Age/duration of feeding practices | | | | | | |
| | | Current breastfeeding | 0.093 (0.907) | 0.225 (0.763) | 3.795 (0.997) | 0.256 (0.999) | 5.523 (0.997) | 0.3733 (0.999) |
| | Unhealthy household environment and inadequate health services[$] | **Baseline determinants** | | | | | | |
| | | Access to drinking water[%] | -0.269 (0.391) | -0.330 (0.291) | -0.764 (0.050)* | -0.743 (0.061) | 0.624 (0.142) | 0.604 (0.164) |
| | | Access to any sanitation[%] | -0.223 (0.201) | -0.207 (0.236) | -0.365 (0.095) | -0.392 (0.079) | 0.185 (0.441) | 0.159 (0.517) |
| | | Access to any hygiene facilities[%] | -0.099 (0.590) | -0.150 (0.415) | -0.082 (0.723) | -0.062 (0.794) | 0.214 (0.394) | 0.200 (0.437) |
| | | Handwashing after cleaning a baby's bottom/changing a baby's nappy | -0.256 (0.215) | -0.226 (0.270) | -0.152 (0.557) | -0.173 (0.511) | 0.165 (0.563) | 0.133 (0.646) |
| | | Handwashing before preparing/handling food | 0.121 (0.675) | 0.139 (0.624) | -0.239 (0.507) | -0.250 (0.491) | 0.118 (0.765) | 0.094 (0.813) |
| | | Handwashing before feeding an infant/eating | -0.218 (0.280) | -0.220 (0.282) | -0.147 (0.561) | -0.173 (0.511) | -0.058 (0.834) | -0.123 (0.670) |
| | | Handwashing after handling raw food | -0.066 (0.669) | -0.033 (0.827) | 0.137 (0.478) | 0.124 (0.526) | -0.289 (0.172) | -0.312 (0.145) |
| | | Handwashing after handling garbage | -0.088 (0.620) | -0.076 (0.663) | -0.085 (0.700) | -0.091 (0.683) | -0.238 (0.326) | -0.247 (0.311) |
| | | Handwashing after field work | -0.356 (0.072) | -0.304 (0.122) | -0.046 (0.852) | -0.068 (0.788) | -0.390 (0.153) | -0.403 (0.146) |
| | | Handwashing after handling livestock | -0.053 (0.729) | -0.076 (0.620) | 0.169 (0.380) | 0.177 (0.367) | 0.036 (0.866) | 0.023 (0.914) |
| | | Handwashing with Soap and water | -0.055 (0.759) | -0.103 (0.570) | -0.387 (0.081) | -0.383 (0.093) | -0.100 (0.687) | -0.125 (0.622) |
| | | Mother using soap to wash hands | -0.068 (0.677) | -0.073 (0.648) | -0.469 (0.020) | -0.471 (0.020)* | -0.047 (0.835) | -0.056 (0.805) |
| | | Cleaning infants, post-defecation | 0.024 (0.894) | 0.040 (0.818) | -0.021 (0.925) | -0.030 (0.894) | 0.323 (0.181) | 0.307 (0.208) |
| | | Handwashing after cleaning infants | -0.071 (0.786) | 0.065 (0.810) | -0.134 (0.671) | -0.207 (0.533) | -0.016 (0.963) | -0.055 (0.881) |
| | | Disposal of infant's stool, post-defecation[%] | 0.141 (0.353) | 0.211 (0.164) | 0.081 (0.669) | 0.063 (0.747) | 0.157 (0.452) | 0.162 (0.450) |
| | | **Time-varying determinants** | | | | | | |
| | | Putting soil/animal feces in mouth | 1.103 (0.000)* | 0.461(0.028)* | 0.730 (0.000)* | -0.369 (0.010)* | 0.694 (0.000)* | -0.027 (0.914) |
| | | Preventing putting soil/animal feces in mouth | 0.924 (0.000)* | -0.066 (0.745) | 0.985 (0.000)* | 0.235 (0.115) | 0.796 (0.000)* | 0.208 (0.405) |
| | | Drinking from a bottle with nipple | 0.844 (0.000)* | -0.067 (0.729) | 0.844 (0.000)* | -0.076 (0.588) | 0.611 (0.000)* | -0.073 (0.749) |
| Control measures | | **Time-varying determinants** | | | | | | |
| | | Any antibiotic use | 0.252 (0.258) | -0.330 (0.123) | 1.270 (0.000)* | 0.348 (0.224) | 0.519 (0.039)* | 0.142 (0.578) |

| | | | | | | | | |
|---|---|---|---|---|---|---|---|---|
| Risks | Exposure to enteric pathogens[@] | **Baseline determinants** | | | | | | |
| | | Cattle count | 0.043 (0.513) | 0.052 (0.428) | 0.069 (0.396) | 0.067 (0.425) | 0.039 (0.663) | 0.023 (0.807) |
| | | Chicken count | 0.009 (0.689) | 0.003 (0.879) | 0.036 (0.207) | 0.039 (0.181) | -0.007 (0.813) | -0.007 (0.822) |
| | | Goat count | 0.007 (0.873) | 0.007 (0.875) | -0.013 (0.802) | -0.014 (0.791) | -0.022 (0.708) | -0.026 (0.653) |
| | | Sheep count | -0.075 (0.072) | -0.075 (0.067)* | 0.011 (0.831) | 0.011 (0.843) | -0.041 (0.483) | -0.046 (0.433) |
| | | Chicken daytime location risk score[%] | 0.466 (0.002)* | 0.443 (0.004)* | 0.070 (0.715) | 0.086 (0.664) | 0.027 (0.900) | 0.010 (0.963) |
| | | Chicken nighttime location risk score[%] | 0.412 (0.006)* | 0.395 (0.010)* | -0.087 (0.647) | -0.083 (0.675) | 0.111 (0.595) | 0.094 (0.667) |
| | | Cattle daytime location risk score[%] | 0.187 (0.291) | 0.214 (0.219) | 0.114 (0.607) | 0.107 (0.633) | -0.119 (0.608) | -0.116 (0.620) |
| | | Cattle nighttime location risk score[%] | 0.238 (0.118) | 0.270 (0.070) | 0.172 (0.369) | 0.163 (0.401) | 0.111 (0.599) | 0.087 (0.682) |
| | | Sheep daytime location risk score[%] | -0.312 (0.106) | -0.320 (0.095) | 0.166 (0.493) | 0.176 (0.476) | -0.565 (0.033) | -0.546 (0.042)* |
| | | Sheep nighttime location risk score[%] | -0.383 (0.011)* | -0.399(0.008)* | -0.082 (0.667) | -0.087 (0.655) | -0.409(0.049)* | -0.445 (0.036)* |
| | | Goat daytime location risk score[%] | -0.124 (0.440) | -0.086 (0.588) | -0.031 (0.878) | -0.047 (0.815) | -0.311 (0.156) | -0.332 (0.134) |
| | | Goat nighttime location risk score[%] | 0.101 (0.508) | 0.070 (0.646) | 0.000 (0.999) | 0.009 (0.964) | 0.013 (0.952) | 0.003 (0.991) |
| | | Collection of livestock waste | 0.219 (0.360) | 0.192 (0.425) | -0.028 (0.925) | -0.025 (0.934) | -0.482 (0.142) | -0.544 (0.106) |
| | | Livestock dropping present in household/homestead | 0.229 (0.137) | 0.217 (0.152) | 0.256 (0.184) | 0.262 (0.177) | 0.234 (0.271) | 0.242 (0.256) |
| | | Slaughtering owned livestock | -0.262 (0.084) | -0.328 (0.030) | -0.016 (0.935) | 0.004 (0.986) | -0.118 (0.574) | -0.117 (0.584) |
| | | Handwashing related to slaughtering | 0.058 (0.784) | 0.126 (0.530) | -0.312 (0.315) | -0.304 (0.342) | 0.314 (0.322) | 0.303 (0.352) |
| | | **Time-varying determinants** | | | | | | |
| | | Raw milk consumption | 1.677 (0.000)* | 0.962 (0.002)* | 1.904 (0.000)* | 0.487 (0.238) | 0.608 (0.107) | 0.041 (0.913) |
| | | Crawling where animal droppings might be present | 1.145 (0.000)* | 0.597 (0.007)* | 2.091 (0.000) | 0.828 (0.004) | 0.843 (0.000)* | 0.227 (0.395) |
| | | Physical contact with livestock | 1.090 (0.000)* | 0.412 (0.034)* | 1.962 (0.000)* | 0.694 (0.006)* | 0.723 (0.000)* | 0.064 (0.789) |
| Benefits | Food production | **Time-varying determinant** | | | | | | |
| | | ASF[#] consumption | 0.303 (0.129) | 0.246 (0.196) | -0.169 (0.567) | -0.354 (0.210) | -0.034 (0.887) | -0.078 (0.730) |
| | Livestock income | **Baseline determinants** | | | | | | |
| | | Total annual income quartile from selling livestock | -0.080 (0.600) | -0.092 (0.554) | 0.148 (0.435) | 0.152 (0.441) | -0.192 (0.357) | -0.249 (0.250) |
| | | TLU[#] | 0.012 (0.885) | 0.027 (0.749) | 0.090 (0.384) | 0.086 (0.424) | 0.018 (0.876) | -0.007 (0.954) |
| Basic causes | Social/cultural | **Baseline determinants** | | | | | | |
| | | Residents in the home (<6 children) | -0.336 (0.205) | -0.242(0.379) | -0.063 (0.759) | -0.109 (0.612) | 0.249 (0.205) | 0.248 (0.212) |
| | Economics/livelihood | **Baseline determinants** | | | | | | |
| | | On-farm as primary livelihood | 0.344 (0.233) | 0.226 (0.436) | 0.094 (0.794) | 0.147 (0.694) | 0.578 (0.144) | 0.609 (0.135) |
| | | Asset quartile | -0.097 (0.522) | 0.085 (0.229) | -0.073 (0.716) | -0.068 (0.735) | -0.167 (0.367) | -0.165 (0.374) |
| | Human capital | **Baseline determinants** | | | | | | |
| | | Mother's age (years) | 0.029 (0.912) | 0.129 (0.632) | -0.60 (0.769) | -0.098 (0.646) | 0.184 (0.341) | 0.174 (0.373) |
| | | Mother attended school | -0.075 (0.799) | -0.216 (0.486) | -0.229(0.342) | -0.184 (0.449) | 0.003 (0.987) | 0.001 (0.995) |
| | Basic demographics | **Baseline determinants** | | | | | | |
| | | Infant being female | 0.612 (0.022)* | 0.608 (0.023)* | -0.139 (0.489) | -0.137 (0.498) | -0.065 (0.724) | -0.059 (0.749) |

| | | | | | | | | |
|---|---|---|---|---|---|---|---|---|
| | | Infant Birth order (<4) | -0.250 (0.340) | -0.149 (0.583) | -0.192 (0.357) | -0.256 (0.251) | 0.293 (0.143) | 0.289 (0.153) |
| | | **Time-varying determinants** | | | | | | |
| | | Infant's age | | | | | | |
| | | Grp(Q#) 4 vs. Grp(Q) 1 | 1.245 (0.000)* | - | 2.215 (0.000)* | - | 1.041 (0.000)* | - |
| | | Grp(Q) 3 vs. Grp(Q) 1 | 0.938 (0.000)* | - | 1.911 (0.000)* | - | 0.375 (0.026)* | - |
| | | Grp(Q) 2 vs. Grp(Q) 1 | 0.363 (0.014)* | - | 0.484 (0.020)* | - | 0.118 (0.483) | - |

[$]For baseline determinants, infant feeding indicators shortly after birth were associated with *Campylobacter* load in month one of age; other determinants were associated with the average *Campylobacter* load of the whole follow-up period12. For time-varying (longitudinal) determinants, each age quartile's average Campylobacter load was regressed on each determinant's concurrent proportion/average or an average for the HFIAS score or the *Campylobacter* load.
[%]Reference group: for binary determinants, the status of absence was treated as the reference; WaSH ladders were binarized in regressions and the categories of unimproved, open defecation, and no facility were respectively treated as reference groups for the *drinking water*, *sanitation*, and *hygiene* ladders, and other categories of each WaSH ladders were merged into a non-reference category for regressions; For *disposal of infant's stool, post-defecation*, we combined the categories of *Left in the open*, *Buried*, *Thrown into garbage* as the non-reference group, and the other three categories as the reference group in the multiple-determinant model. Definition of each category of livestock location risk score – 0: household did not have the livestock species or kept them outside the house; 1: household kept the livestock species inside the house and confined them; 2: household kept the livestock species inside the house but did not confine them.
[^]Adjusted for sex and socioeconomic status (indicated by a binary variable of household asset) at baseline, and age was further adjusted through random intercepts in linear-mixed models for time-varying determinants. [*]p<0.05
